# Proton boron capture therapy (PBCT) induces cell death and mitophagy in a heterotopic glioblastoma model

**DOI:** 10.1038/s42003-023-04770-w

**Published:** 2023-04-08

**Authors:** Francesco Paolo Cammarata, Filippo Torrisi, Nunzio Vicario, Valentina Bravatà, Alessandro Stefano, Lucia Salvatorelli, Simona D’Aprile, Pierangela Giustetto, Giusi Irma Forte, Luigi Minafra, Marco Calvaruso, Selene Richiusa, Giuseppe Antonio Pablo Cirrone, Giada Petringa, Giuseppe Broggi, Sebastiano Cosentino, Fabrizio Scopelliti, Gaetano Magro, Danilo Porro, Massimo Libra, Massimo Ippolito, Giorgio Russo, Rosalba Parenti, Giacomo Cuttone

**Affiliations:** 1 Institute of Molecular Bioimaging and Physiology, National Research Council, IBFM-CNR Cefalù, Italy; 2 National Institute for Nuclear Physics, Laboratori Nazionali del Sud, INFN-LNS Catania, Italy; 3 Department of Biomedical and Biotechnological Sciences, University of Catania, Catania, Italy; 4 Molecular Preclinical and Translational Imaging Research Center - IMPRonTe, University of Catania, Catania, Italy; 5 Department G.F. Ingrassia, Azienda Ospedaliero-Universitaria “Policlinico-Vittorio Emanuele” Anatomic Pathology, University of Catania, Catania, Italy; 6 Nuclear Medicine Department, Cannizzaro Hospital, Catania, Italy; 7 Radiopharmacy Laboratory Nuclear Medicine Department, Cannizzaro Hospital, Catania, Italy

**Keywords:** Cancer therapy, Cancer imaging, CNS cancer

## Abstract

Despite aggressive therapeutic regimens, glioblastoma (GBM) represents a deadly brain tumor with significant aggressiveness, radioresistance and chemoresistance, leading to dismal prognosis. Hypoxic microenvironment, which characterizes GBM, is associated with reduced therapeutic effectiveness. Moreover, current irradiation approaches are limited by uncertain tumor delineation and severe side effects that comprehensively lead to unsuccessful treatment and to a worsening of the quality of life of GBM patients. Proton beam offers the opportunity of reduced side effects and a depth-dose profile, which, unfortunately, are coupled with low relative biological effectiveness (RBE). The use of radiosensitizing agents, such as boron-containing molecules, enhances proton RBE and increases the effectiveness on proton beam-hit targets. We report a first preclinical evaluation of proton boron capture therapy (PBCT) in a preclinical model of GBM analyzed via μ-positron emission tomography/computed tomography (μPET-CT) assisted live imaging, finding a significant increased therapeutic effectiveness of PBCT versus proton coupled with an increased cell death and mitophagy. Our work supports PBCT and radiosensitizing agents as a scalable strategy to treat GBM exploiting ballistic advances of proton beam and increasing therapeutic effectiveness and quality of life in GBM patients.

## Abstract (web summary)

Proton boron capture therapy (PBCT) in a preclinical glioblastoma model exhibits increased therapeutic effectiveness with an increased cell death and mitophagy, supporting its use as a scalable strategy to treat glioblastoma.

## Introduction

Improvements of non-invasive radiotherapy treatments techniques in neuro-oncology practice are essential especially for brain tumors due to the need to accurately delineate structures of interest based on multimodal imaging and the vulnerability of healthy organs at risk^1,2^. Despite the advancement of targeted irradiation technology systems, such as stereotactic, intensity-modulated, image-guided radiotherapy and the large advances in tumor volume delineation strategies, to date biomedical approaches are unsuccessful for the treatment of Glioblastoma (GBM), which belongs to the category of the more aggressive, radioresistant and hypoxic tumors, characterized by a dismal prognosis both in terms of median survival and progression free survival^3,4^. In particular, physiological hypoxia is easily established into the GBM tumor, due to its rapid proliferation, promoting radioresistance and chemoresistance to treatments^5^.

Current therapeutic strategy relies on surgical resection, temozolomide treatment and photons, X- or Gamma-rays, conventional radiotherapy (60 Gy dose in 30 fractions of 2 Gy each)^6,7^. Notwithstanding such a robust therapeutic regimen, we are currently unable to fully counteract tumor growth and recurrencies, beyond the highly impacting therapy-derived side effects^8^. As such, the clinical need to improve the cell-killing efficacy with ionizing radiation becomes undisputable; in this regard, alternative sources of ionizing radiation are currently under investigation in order to maximize the risk-benefit ratio by combining high linear energy transfer (LET) features and precision dose distribution of heavy and charged particles. Proton therapy (PT) may represent a promising approach for brain tumors, since protons show a favorable depth-dose distribution by releasing the utmost amount of energy at a definite depth along their track, corresponding to the so-called Bragg peak^9–11^. This allows to reduce radiation dose and off-target effects on healthy tissues, particularly affected in the brain^12,13^. However, such advantages in using protons as source of ionizing radiation are coupled with their low relative biological effectiveness (RBE) in killing tumor cells^11,14,15^. Therefore, the benefit offered by the typical depth-dose profile of protons, can be exploited mainly to reduce side effects in low-grade pediatric glioma or in the re-irradiation of recurrent high-grade gliomas, but it is not effective in counteracting tumor progression of radioresistant and hypoxic tumors such as GBM^16,17^. In such an aggressive and radioresistant tumor, strategies to enhance proton RBE become a crucial challenge in the field.

Here we report a first preclinical evaluation of a promising radiation technique, called Proton Boron Capture Therapy (PBCT) in a heterotopic GBM mouse model. PBCT is a novel therapeutic approach aimed at producing a physical-driven radiosensitization using protons. It exploits a nuclear fusion reaction between low-energy protons and 11B atoms, i.e. p+11B→3α (p-B), then enhancing proton biological effectiveness, due to the α-particles release that are supposed to generate DNA double-strand breaks across Spread-Out Bragg Peak (SOBP). This approach allows the radiosensitization to be confined exclusively to the target hit by the proton beam^18^.

To date, only Monte Carlo studies for dosimetric simulation have been performed and promising in vitro results have been obtained by evaluating cell damages induced by PBCT^18–20^. We previously performed a proof-of-concept experiment using PBCT in prostate cancer cell line DU145 and human mammary epithelial cells MCF-10A and we found that cellular lethality was significantly improved by irradiation of cells that had been pre-treated with boron phenylalanine (BPA) as a boron carrier^18^.

Herein, we investigate the PBCT in a heterotopic model of GBM, analyzing pathophysiological and molecular effects in response to this innovative in vivo therapeutic approach. We first validated the reliability of our GBM model by multimodal ultrasound and photoacoustic imaging technique, allowing the assessment of the tumor oxygenation over the tumor growth. Then, metabolic changes after PBCT treatment were detected by μ-positron emission tomography/computed tomography (µPET-CT) assisted scanning. Finally, pathological analyses associated to RNA-seq were performed to examine tissue biological effects and molecular pathways after PBCT treatment.

## Results

### Oxygen saturation is reduced overtime in heterotopic GBM model

**Fig. 1:**
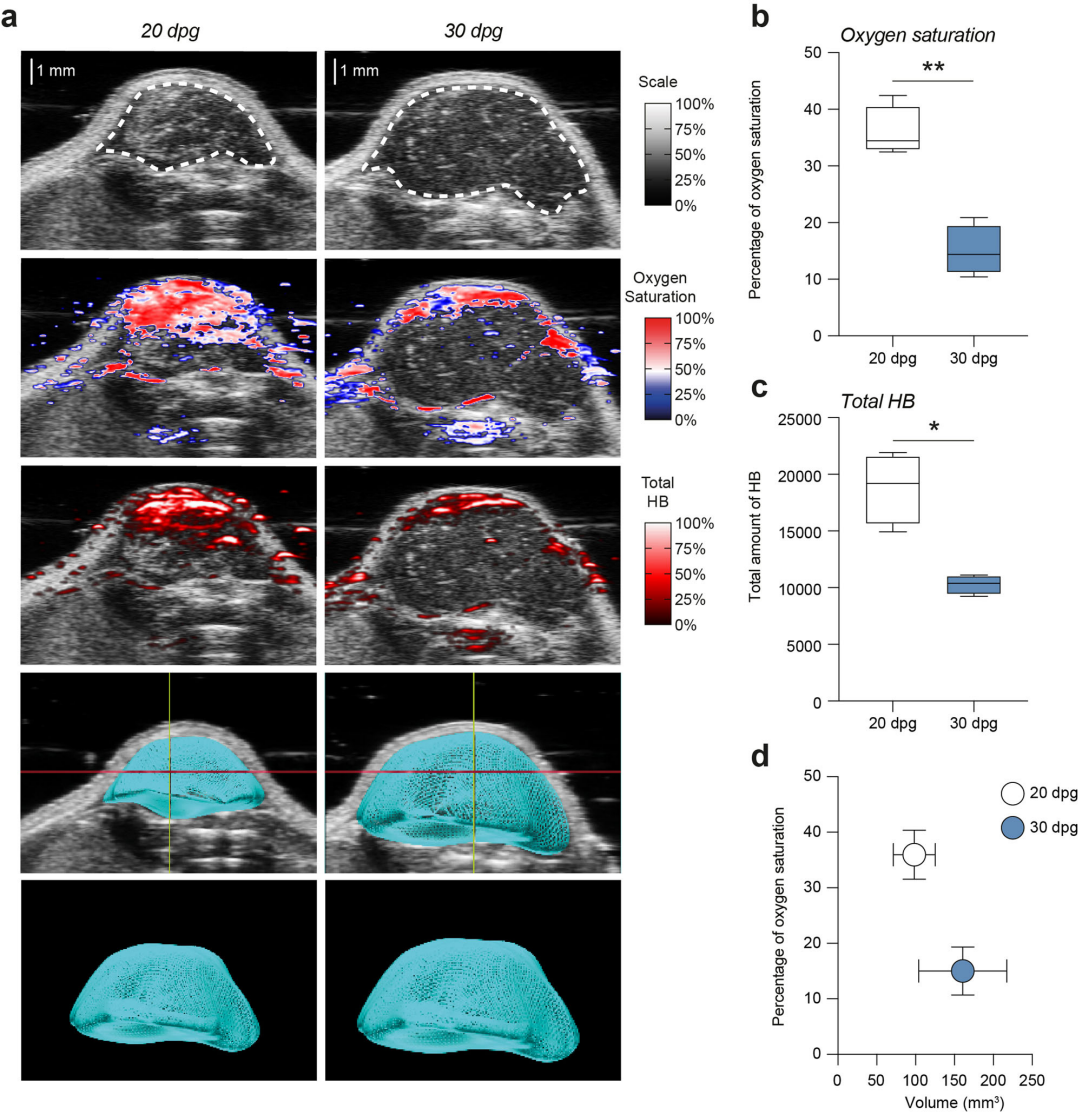
Tumor oxygenation and hemoglobin are reduced overtime and inversely correlate with tumor growth. **a** Ultrasound-photoacoustic assisted imaging of heterotopic GBM showing representative 2D frames tumor mass, oxygen saturation, total hemoglobin and 3D reconstruction of tumor mass at 20 and 30 dpg. **b**–**c** Quantification of oxygen saturation and total hemoglobin levels within the tumor mass at 20 (white boxes and white circle) and 30 (blue boxes and blue circle) dpg. **d** Biplot of the percentage of oxygen saturation and tumor volume expressed in mm^3^. *p-value < 0.05 and **p-value<0.01. HB hemoglobin, dpg: days post-graft.

As known, intra-tumor oxygen levels in vivo are reduced overtime and inversely correlate with tumor growth, establishing physiological hypoxia and possible radioresistance^5^. In order to analyze this aspect, we established a heterotopic GBM model using U-87 MG cells grafted subcutaneously, analyzing oxygen saturation, total hemoglobin (HB) and tumor growth in the untreated mice and in the mock proton group at 20 and 30dpg. Using an ultrasound/photoacoustic-assisted imaging we observed a significant reduction of the percentage of oxygen saturation at 30dpg as compared to 20dpg (Fig. 1a, b). Such evidence was coupled with a significant reduction of total HB at 30dpg versus 20dpg (Fig. 1a, c), indicating reduced oxygenation and vascularization in heterotopic GBM overtime, which negatively correlate with tumor growth (Supplementary Fig. 1, 2 and Fig. 1d).

### Proton boron capture therapy reduces total lesion glycolysis in vivo

**Fig. 2:**
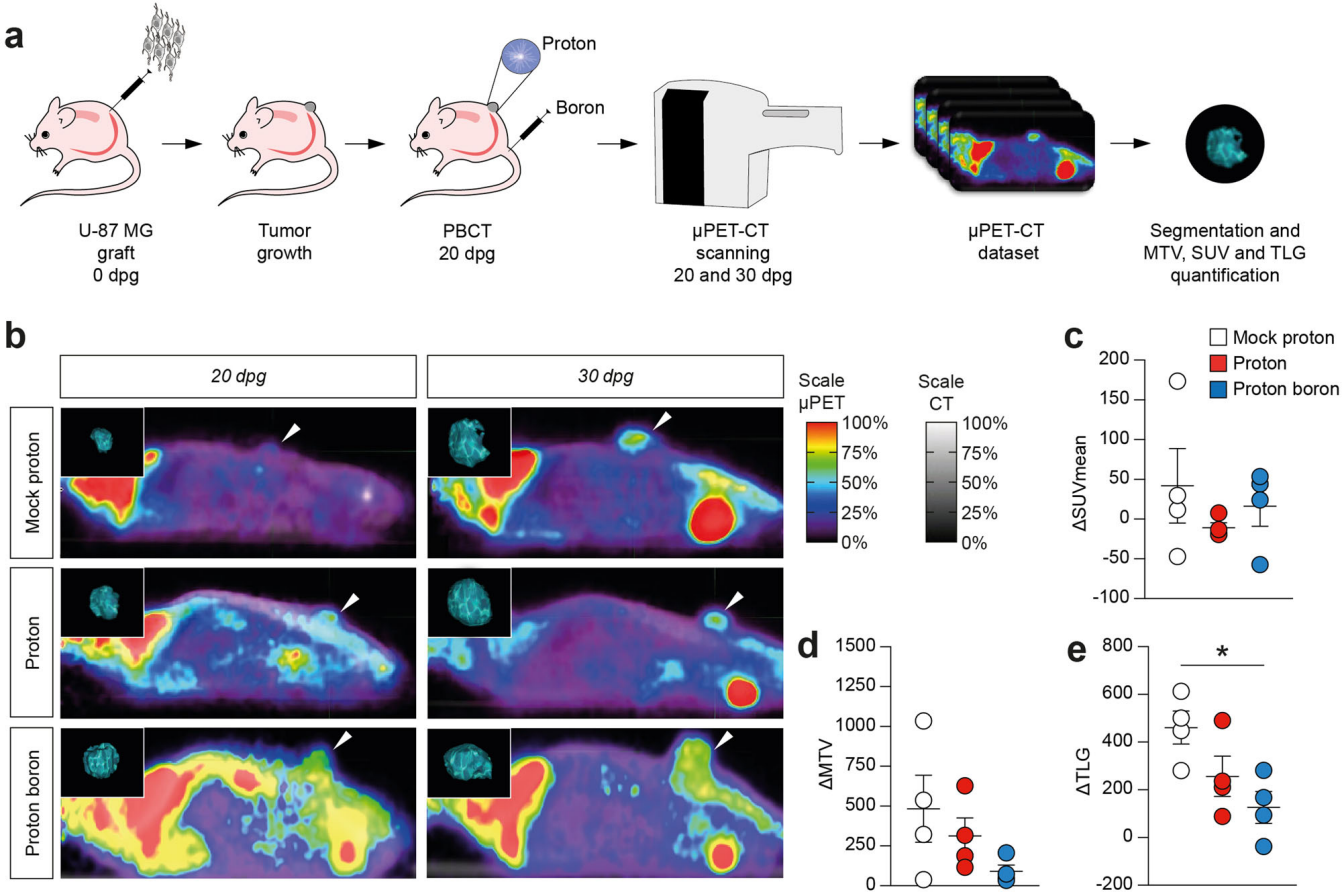
Proton irradiation and proton boron capture therapy induce a reduction of SUV at 30dpg. **a** Experimental setting for GBM induction, proton irradiation and FDG PET scanning. **b** Representative FDG PET images of mock proton, proton-treated and proton boron-treated mice. **c**–**e** Quantification of ΔSUV, ΔMTV and ΔTLG between 20 and 30dpg in mock proton (white circles), proton-treated (red circles) and proton boron-treated (blue circles) mice. *p-value < 0.05. Dpg days post-graft, SUV standardized uptake value, MTV metabolic tumor volume, TLG total lesion glycolysis.

In order to evaluate the effect of PBCT on an in vivo GBM model, we established a preclinical experimental setting to either perform proton irradiation and to analyze, using a μPET-CT assisted live imaging platform, clinically scalable PET parameters (Fig. 2a and Supplementary Fig. 3).

All MTVs, in both 20 and 30dpg, were found spherical and with a uniform radioactive uptake: the spherical condition was satisfied when the percentage difference between the maximum and minimum diameter of the lesion compared to the maximum diameter was less than 50%, while lesion uniformity was satisfied if one single peak, within the lesion, was qualitatively detected on the histogram. Therefore, the PVE correction method was applied to our SUV data.

Since the TLG provides a simultaneous estimation of MTV and SUV for volumetric and metabolic information respectively, the lower its value, the greater the metabolic response to treatment. As a result, both ΔTLG and ΔMTV indicate a better response in combined treated mice in comparison with mock proton and proton groups (Fig. 2b–e).

### Proton boron capture therapy induces apoptosis and cell death

**Fig. 3:**
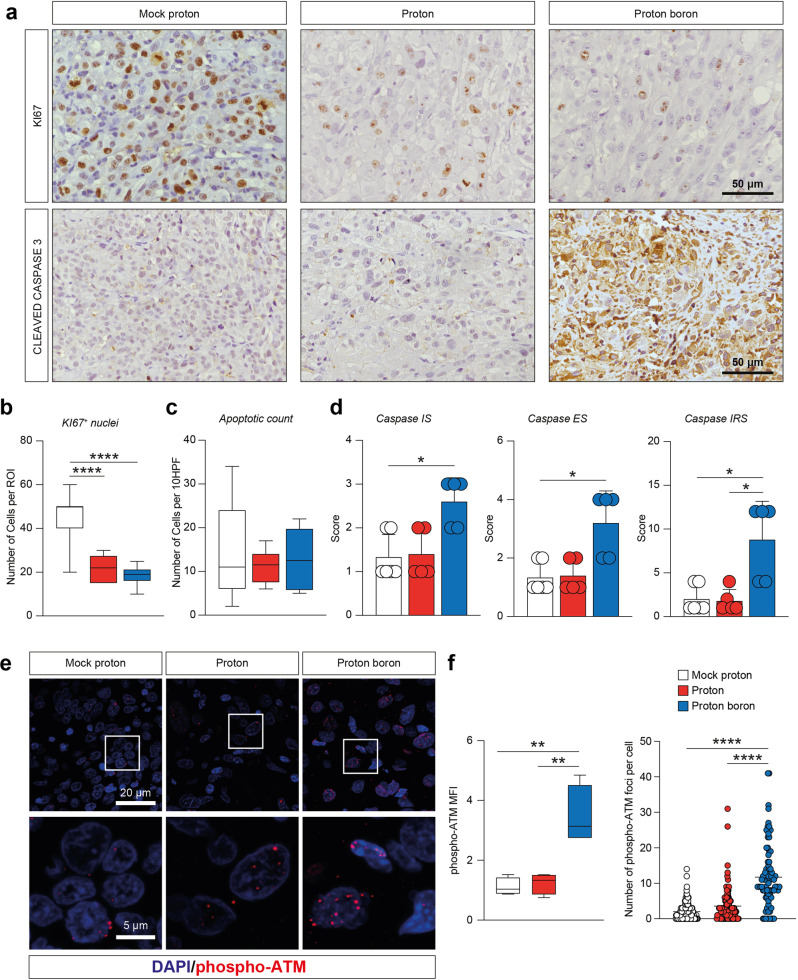
Proton boron capture therapy induces similar cell death effects as compared to proton irradiation but a higher caspases signal. **a** Representative images of KI67 nuclei and caspases in GBM tumor sections derived from mock proton, proton-treated and proton boron-treated mice. **b**–**d** Quantification of KI67 positive cells, apoptotic count, and percentage of immunopositivity cells for caspases in GBM tumor sections derived from mock proton (white boxes and white circles), proton-treated (red boxes and red circles) and proton boron-treated (blue boxes and blue circles) mice. **e**, **f** Representative pictures and quantification of the MFI and number of foci per cell of phospho-ATM staining in mock proton (white boxes and white circles), proton-treated (red boxes and red circles) and proton boron-treated (blue boxes and blue circles) mice. Nuclei are stained with DAPI. *p-value < 0.05, **p-value < 0.01 and ****p-value < 0.0001. IS Intensity score, ES Extent Score, IRS intensity reactivity score, MFI mean fluorescence intensity.

We then moved to analyze immunohistochemically isolated grafts. We stained GBM tissue sections with the proliferation marker KI67 and apoptotic marker caspase 3 (Fig. 3a).

Our analysis revealed that the mock proton group showed a significantly higher mitotic index and a higher proportion of KI67 positive nuclei, as compared to either proton treated and proton boron treated group (Fig. 3b and Supplementary Fig. 4). Importantly, even if we did not observe significant changes in the total apoptotic count, we found a significant increase in caspase intensity and immunopositive cells (Fig. 3c, d).

Such a significant increase in apoptosis was confirmed by IRS score, that was found to be about 5 folds increased in proton boron group as compared to proton and to mock proton (Fig. 3d).

In order to verify the potential DNA damage induced by protons and the effect of boron in enhancing subtherapeutic dose of protons, we performed an immunofluorescence staining for phospho-ATM, which initiates the DNA-damage response. Our data suggest a significant increase of the overall phospho-ATM MFI in proton boron versus proton and versus mock proton group (Fig. 3e, f). These data were confirmed quantifying the total number of phospho-ATM foci per cell, finding a strong activation in proton boron as compared to control groups (Fig. 3f).

**Fig. 4:**
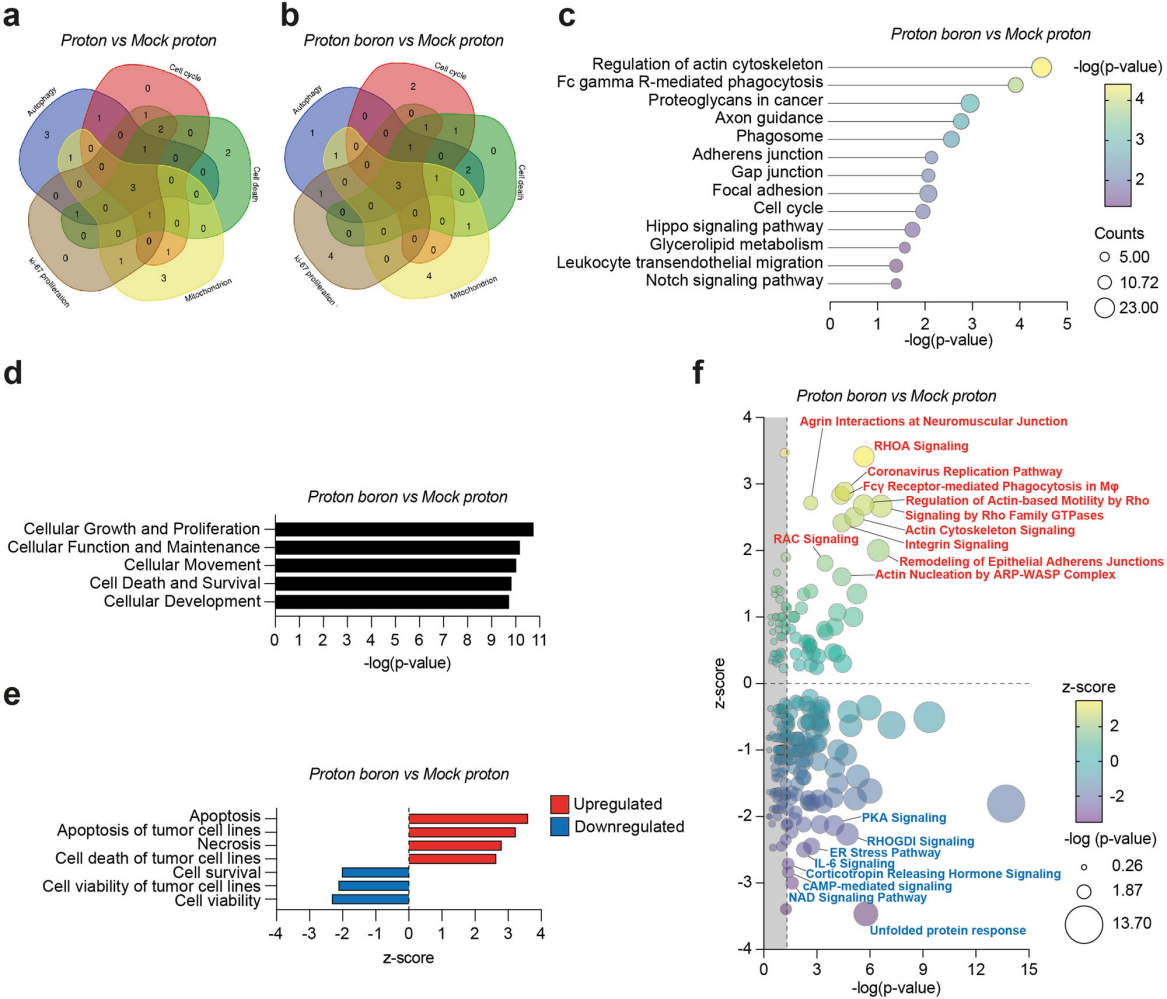
PBCT induces cell death upregulation and cell viability downregulation pathways in association with cytoskeleton remodeling and cellular communication. **a**, **b** Functional enrichments analysis of differentially expressed genes with Venn diagrams of gene expression profiles in proton vs mock proton and in proton boron vs mock proton. **c** Specific biological pathways analysis by DAVID software of upregulated transcripts in which dots are key colored according to their – log(*p*-value) and their dimensions are proportioned to counts number. **d**, **e** Specific statistically significant pathways analysis by IPA software and detailed IPA analysis of death/survival balance. **f** Data enrichment analysis with most statistically and biologically relevant pathway in GBM tumor sample derived from mock proton versus proton boron treated mice in which dots are key colored according to their z-score and their dimensions are proportioned to their – log(*p*-value).

We then moved to analyze the differential gene expression modifications by RNA-Seq approach, to study the biological processes activated in Balb/c nude U-87 MG xenografts mice model treated with proton boron. Firstly, we evaluated whether BPA administration was able to induce molecular modifications in proton-treated tumors via unbias RNAseq analyses of PBCT vs proton-treated samples, highlighting differentially expressed genes (DEGs) sets on proton boron-treated mice. The comparative differential gene expression analysis on the proton boron treated versus proton group revealed that a few genes showed a significantly altered expression level as compared to the reference proton-treated sample: 138 differentially expressed genes (DEGs) 109 down-regulated and 29 up-regulated. Moreover, up-regulated transcripts were selected and grouped according to their involvement in specific biological pathways, using the DAVID tool, identified *Rap1* and *PI3K-AKT* pathways as statistically relevant pathways. *Rap1* encodes a protein involved in a complex regulating telomere length, possibly involved in the activation of the senescence process, often induced by IR, contributes to maintaining genome stability by protecting telomeric DNA ends from non-homologous end joining and from homologous recombination^5^. On the other hand, the *PI3K-AKT* pathway was described as able to regulate glucose metabolism, conditioning tumor growth, angiogenesis, and invasion also in GBM disease (Supplementary Data 1)^5^. We then analyzed the Gene Expression Profiles (GEPs) induced by proton and proton boron compared to the mock proton control tumors. In addition, we analyzed the gene expression modifications of selected transcripts involved in the regulation of autophagy, cell cycle, proliferation KI67 related and mitochondrial activities, in order to evaluate the cell death/survival balance (Fig. 4a, b).

In tumors exposed to proton irradiation the following 3 common genes were able to regulate the cellular processes as shown in Fig. 4a: forkhead box O1 (*FOXO1*, the specific function of this gene has not yet been determined; however, it may play a role in myogenic growth and differentiation); carbonic anhydrase 9 (*CA9*, involved in respiration, acid-base balance and may be involved in cell proliferation and transformation) and GLI family zinc finger 1 (*GLI1*, a transcription factor that regulates stem cell proliferation). In tumors exposed to proton boron three other genes were commonly deregulated during the selected processes described in Fig. 4b: Fos proto-oncogene (*FOS*, implicated as regulators of cell proliferation, differentiation, and transformation also in response to irradiation as several describe in literature also by our group); BCL2 antagonist/killer 1 (*BAK1*, which belongs to the BCL2 protein family, localizes to mitochondria able to induce apoptosis) and zinc finger protein 594 (*ZNF594*, predicted to enable DNA-binding transcription activator activity).

In order to analyze genes and intracellular pathways activated after proton boron treatments in the U-87 MG xenografts mice model, we performed a comparative differential gene expression analysis on the proton boron treated versus mock proton group, which revealed that a conspicuous number of genes showed a significantly altered expression levels as compared to the reference mock proton tumors. Our analysis revealed 1669 differentially expressed genes (DEGs; 969 down-regulated and 700 up-regulated). Moreover, up-regulated transcripts were selected and grouped according to their involvement in specific biological pathways using the DAVID tool (Fig. 4c).

Five among the 13 statistically relevant pathways activated in the proton boron group, were strictly involved in binding and communication with other cells (i.e. regulation of actin cytoskeleton; axon guidance; adherens junction; gap junction, and focal adhesion; Fig. 4c).

We then moved to perform an IPA analysis in order to identify dysregulated pathways in proton boron versus mock proton groups (Fig. 4d, e). Summarizing, in proton boron group a down-regulation of cells survival, growth, and viability and on the other hand an up-regulation of cell death, particularly of apoptosis and necrosis are evident.

Finally, in order to enrich data interpretation, Next Generation Sequencing data coming from proton boron group were analyzed by using IPA pathways analysis tool showing the top-statistically deregulated pathway in this group (Fig. 4f). Modification of cell structures sustained by the arrangement of the cytoskeleton (i.e. up-regulation of actin cytoskeleton signaling and motility also mediated by RAS homolog family member A, *RHOA*), as well as the stimulation of cell communication (i.e. up-regulation of integrine signaling, adherens and neuromuscular junctions), above described by DAVID analysis, were confirmed by IPA. Among the upregulated pathways selected by IPA, we found that proton boron also induces activation of *RAC* signaling (Fig. 4f).

On the other hand, Fig. 4f also shows additional downregulated pathways in proton boron group involved in: calcium homeostasis (i.e. PKA and cAMP-mediated signaling), hormone pathways (ER and Corticotropin signaling), regulation of redox status (NAD signaling), and inflammation (interleukin 6, IL6 signaling).

### Proton boron capture therapy increases mitochondrial autophagy

In order to highlight the activation of the cell death process, we better explored mitochondrial autophagy by immunofluorescence analysis on ex vivo tissue sections of heterotopic GBM of mock proton, proton irradiated and proton boron treated tumors.

**Fig. 5:**
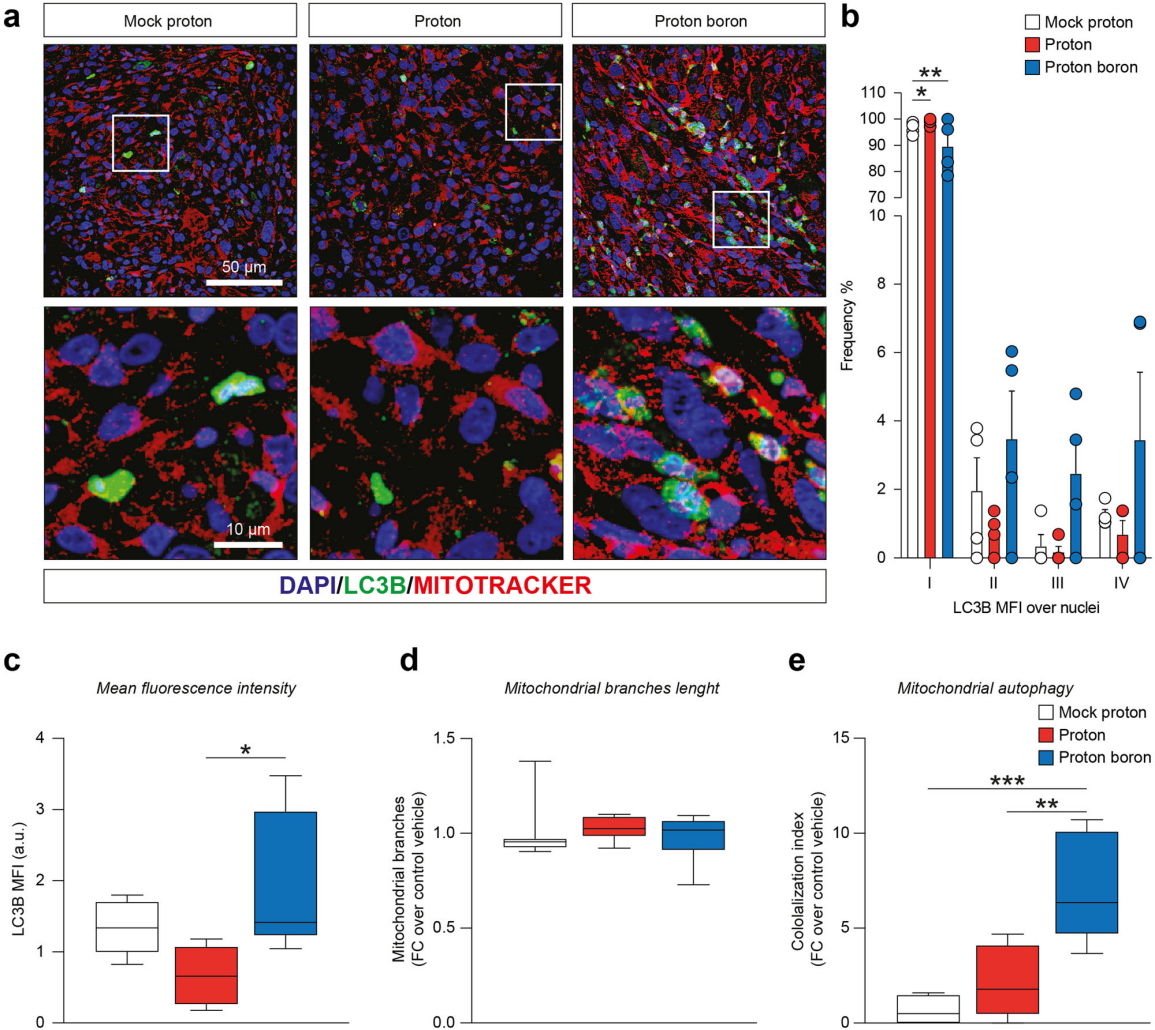
PCBT increases the structural protein of autophagosomal membranes LC3B and mitophagy response. **a** Representative confocal-assisted immunofluorescence pictures of LC3B and mitotraker in GBM tumor sections derived from mock proton, proton-treated and proton boron-treated mice; **b**–**e** Quantification of the intensity range (I and II = low; III = medium; IV = high) frequency of LC3B, LC3B MFI cells, mitochondrial branches and LC3B/mitotraker colocalization in GBM tumor sections derived from mock proton (white dots and white boxes), proton-treated (red dots and red boxes) and proton boron-treated mice (blue dots and blue boxes). *p-value < 0.05, **p-value < 0.01, ***p-value < 0.001.

Our analysis revealed that proton boron group showed a significant increase of nuclear LC3B MFI frequency as compared to the other groups (Fig. 5a, b). Importantly, these evidences were coupled with an overall increase of LC3B MFI in proton boron group of about 2 folds as compared to proton irradiated group (Fig. 5c).

We then moved to analyze mitochondrial branches length via a mitotracker staining finding no significant differences among analyzed groups (Fig. 5d). Notably, we observed a significant increase of colocalization index between LC3B and mitochondrial branches length in proton boron treated tumors as compared to either mock proton and proton group (Fig. 5e). Our analysis suggests that proton boron capture therapy strongly increases tumor autophagy and mitophagy, thus supporting proton boron combinatorial approach for GBM therapy.

## Discussion

Despite aggressive therapeutic regimes combining surgical resection, temozolomide and radiotherapy, GBM is still the most aggressive and lethal brain tumor^21^. Research in the field is focusing on increasing the effectiveness of treatments by limiting their highly impacting side effects. New sources of ionizing radiations are of particular interest given the urgent need to develop either personalized treatment and precise dose distribution, increasing therapeutic effectiveness and quality of life. The singular physical and radiobiological characteristics of high-energy charged particles increasingly confirm the therapeutic use of proton beams as an alternative to X-ray-based conventional radiotherapy. Protons have the advantage of a sharp modulation of irradiation defined by Bragg peak energy distribution, but provide low RBE as compared to photon beams. Therefore, the need of radiosensitizers, as dose modifying factors enhancing protons effectiveness, become evident^22–24^. Exploitable strategies to increase proton RBE may identify two clinical approaches: i) biological ii) physical. On one hand, biological approaches are based on molecularly targeted drugs and nanoparticles, aimed at blocking specific targets promoting radioresistance or at increasing cell death synergistically with radiations. Promising results have been obtained combining protons with treatments inducing DNA damage breaks or failure repair such as poly ADP-ribose polymerase (PARP) inhibitors, or therapeutic agents arresting signaling pathways involved in cancer malignancy, such as SRC tyrosine kinase inhibitors, but first studies in human have not yet been performed^25–27^. On the other hand, physical approaches use distinctive properties of energy and particles. PBCT aims to exploit boron-releasing molecules to increase protons RBE by exploiting the three short-range high-LET α-particles produced by the reaction between the protons and the boron isotopic ^10^B/^11^B. Such a phenomenon may also be exploited by selective carriers of boron into tumor cells, paving the way for an even more selective approach.

Herein, we sought to evaluate the effects of PBCT in a reductionistic preclinical model as a proof-of-principle in the context of GBM. To do so, we investigated the potential of protons using sub-therapeutic dose (i.e. 10 Gy) to avoid confounding factors related to significant tumor regression due to PT. Such an approach allows us to evaluate the effect of BPA in increasing protons RBE. BPA has been previously tested in deep for its boron carrier properties on preclinical models subjected to the Boron Neutron Capture Therapy (BNCT), revealing its ability to accumulate mainly in the tumor respect to healthy tissues and showing to be safe and nontoxic^28^.

Functional analyses suggest a major efficacy of PBCT as compared to PT alone. In particular, a significantly lower mitotic index and proportion of KI67 positive nuclei were observed both in proton and proton boron groups versus mock proton mice. In addition, PBCT induces a significant increase of cleaved caspase 3 IS, ES and IRS, where the last parameter was found to be increased 5-fold in PBCT treated mice as compared to alone proton irradiated mice, suggesting the PBCT ability to promote more apoptosis than PT, in line with previous studies on in vitro GBM model studies, where the combined treatment was able to significantly increase cell damages^18–20^.

Such an effect is also confirmed by [^18^F]-FDG µPET imaging, which highlighted the reduction of ΔTLG ten days after treatment in the PBCT mice compared to PT. Also, it can be hypothesized that the reduced [^18^F]-FDG uptake, found in the PBCT group, promotes this treatment for its ability to counteract the hypoxia related radioresistance, observed by ultrasound imaging at 30dpg in untreated mice. In fact, it is known that hypoxia triggers the Warburg effect, which increases glycolytic metabolism^5^. Therefore, the finding of a lower metabolic glycolytic activity, through [^18^F]-FDG PET, confirms the superiority of PBCT over PT in counteracting the mechanisms of hypoxic radioresistance. Furthermore, the high ionizing potential of alpha particles generated by the reaction between protons and boron will ensure an oxygen independent effect; hence the resolution of DNA adducts by compounds containing sulfhydryl groups under hypoxic conditions, generated by the indirect effect of water radiolysis would be negligible (oxygen fixation hypothesis). Thus, our results suggest that PBCT induces major therapeutic effects than PT alone counteracting tumor cell survival and proliferation and inducing cell death.

Then, in order to identify more detailed molecular pathways involved in PBCT-mediated effect, we performed an RNA-seq analysis on heterotopic GBM tumors. The arrangement of the cytoskeleton significantly affects tumor formation and growth. Particularly, it was recently described that protons increased cellular plasticity through changes in actin cytoskeleton organization, a process known to be strongly connected to cancer invasion^29^. Similarly, 5 out 13 statistically relevant pathways activated in the proton boron group were strictly involved in cytoskeleton reorganization and cell-cell communications (i.e. regulation of actin cytoskeleton; axon guidance; adherens junction; gap junction, and focal adhesion). These activated pathways in proton-boron GBM, could affect the fate of surrounding cells via bystander mechanisms. Suzuki and colleagues have recently observed lethality and mutagenicity via gap-junction-mediated cell-cell communication in human fibroblast cells after high-LET irradiation exposure^30^, a signalling found activated in PBCT group. Also, Fc gamma R-mediated phagocytosis and phagosome pathways, upregulated in PBCT, were involved in cytoskeletal changes and cell communication. In addition, phagocytosis is tightly linked to the reactive oxygen intermediates and nitric oxide homeostasis after irradiation and to the expression of cytokines, chemokines, and other proteins regulating immunity. These networks clearly emerged as deregulated pathways in PBCT versus mock proton groups^31^. In turn, the activation of the proteoglycans in cancer, leukocyte transendothelial migration, phagosome pathways, have many roles in tumor progression and their activation was also observed in PBCT group^25^. On the other hand, few literature data are available regarding glycerolipid metabolism and its deregulation after ionizing radiation exposure, probably involved in exosome metabolism and cell communication^32^.

The alterations in the filamentous cytoskeleton and altered mechano-transduction related pathways have been described as able to activate Hippo signaling, and thus to enhance the tumor formation, growth, and affect GBM radioresistance^33^. It is worth noticing that Hippo signaling pathway and its role in the maintenance of tissue homeostasis, including upon ionizing radiation-induced damages, is not fully elucidated. Based on our previous results on its activation after proton irradiation in an in vitro model of GBM, we suggested a potential relevance of Hippo signaling, indicating a potential therapeutic application of Hippo signaling inhibitors as an effective anticancer strategy^25^.

Moreover, our findings are also in agreement with previous published evidence on the deregulation of the cell cycle, often associated with ionizing radiation treatment, causing a modification of cell cycle/cell death balance. Indeed, ionizing radiation may activate either pro- and anti-proliferative signal pathways producing an imbalance in survival/apoptosis cell fate^34^. In line with this evidence, it has been well supported the role of Notch signaling, critical for neural cell growth and homeostasis. Targeted therapies against Notch signaling counteract neoplastic glioma cells survival and proliferation^35^. Importantly, recent evidence supports the hypothesis that PBCT in GBM cells induces a Notch signaling activation, able to regulate cell fate through the modulation of autophagy/apoptosis transition^36^. RHO family GTPases have been described as aberrantly regulated in GBM and able to affect cell invasion and tumor progression, via actin remodeling^37–39^. In addition, RHOA acts in tandem with other factors, such as diacylglycerol kinase alpha (DGKα), to induce a resistant mesenchymal phenotype in GBM cells^38^. This scenario could be sustained also by the upregulation of glycerolipid metabolism observed by our DAVID analysis. Fc gamma R-mediated phagocytosis was selected as statistically relevant process induced by PBCT, also by using IPA. Thus, we speculate its key role in cell response to PBCT. RAC signalling, also known as AKT serine/threonine kinase 1 (AKT1) signaling, has been often described as deregulated upon irradiation, including PT. This factor is described as involved in regulating survival/autophagic processes and in the development of chemo- and radio-resistance phenotype of GBM, mediated by AKT-mTOR signaling^40^. PI3K/AKT/mTOR inhibitors in combination with ionizing radiation increase the radiosensitivity of malignant cells to radiation activating autophagy and represent a therapeutic approach of radiochemotherapy in GBM patients^40–42^.

Overall, the GEP analysis in the PBCT group shows the activation of molecular mechanisms induced by radiations, as expected by the fact that PBCT is a physical-driven approach of radiosensitization.

Interestingly, we expanded our investigations on tumor autophagy. Being likely a context-dependent mechanism, we examined a possible involvement of specific cellular components in the autophagic process; the co-localization of autophagic signal with mitochondrial staining revealed that PCBT determined also a mitophagy response. This effect may be implicated in a cytoprotective process, which would ensure a metabolic turnover and reprogramming for the maintenance of functional homeostasis in treated cells. Indeed, it has been demonstrated that autophagic process mediates metabolic reprogramming^43^.

A future perspective of our study is represented by the investigation of the PBCT treatment addressing the question to optimize proton irradiation and GBM tumor models orthotopically. In our previously work, we performed a first experimental proof of PBCT in prostate cancer cell line DU145 and human mammary epithelial MCF-10A cells and we found that cellular lethality was significantly enhanced by irradiating cells that had been pre-treated with sodium borocaptate (BSH)^18^. Herein, we postulate, using a cutting-edge biomedical strategy, a significant therapeutic potential of PBCT for GBM using a reductionistic heterotopic GBM model. Our approach implies some limitations linked to the lack of the typical microenvironment surrounding GBM cells and the potential hampering effects of physiological barriers of the central nervous system. Of note, the correct localization of tumor will make possible to evaluate aspects related to the boron transportation into the tumor cells, considering the selective role of the blood-brain barrier. Moreover, tumoral and stromal cells interaction, especially in GBM, generate a complex tumor microenvironment, which is of critical importance to establish successful therapeutic strategies. The evidence of cell communication and possible bystander mechanisms in response to treatment, will surely benefit of future studies elucidating the interactions between stromal cells and tumor counterpart in response to PBCT. We also would evaluate PBCT in high-grade GBM in relation to other histological and molecular characteristics such as the isocitrate dehydrogenase (IDH) status, which represents a nodal point as referred by the updated classification of gliomas of the World Health Organization^44^. A recent study reported that an increased oncometabolite 2-hydroxyglutarate in IDH mutated high-grade gliomas drives immunosuppression mechanisms by stimulating tryptophan metabolism with an increase of L-type amino acid transporter 1 (LAT1)^45^. These immunometabolism processes could have important consequences related to uptake of borate compounds such as BPA, which being a ^10^B-derivative of tyrosine/phenylalanine is taken up via LAT1 transporter^46,47^. Therefore, future studies could be directed to the evaluation of boron uptake in relation to the molecular characteristics of GBM to optimize the therapeutic boron delivery in tumor cells.

The clinical translation of such innovative radiotherapy treatment requires the evaluation of these radiation-induced mechanisms; moreover, this preclinical study focused on the molecular effects induced by PBCT approach that may provide the identification of further druggable targets leading to additional radiosensitizing therapies.

However, PBCT bio-physical rationale is close to the BNCT approach, which is already included in the clinical setting; as for PBCT, in BNCT, boron-enriched compounds such as BSH and BPA trigger the reaction between thermal neutrons and ^10^B-nuclei to produce high LET radiations composed by one α particle and one ^7^Li ion^48^; however, BNCT showed major limitations related to the difficulties on beam energies standardization among each neutron beam occurred^49^. Among charged and heavy particles for therapeutic use, ions such as carbon ions or hadrons in general share a similar depth–dose profile of protons, but despite their higher LET and RBE, the large cost of carbon ion facilities make difficult their therapeutic application^50,51^. Indeed, according to the newest updates of the Particle Therapy Co-Operative Group (PTCOG), nowadays several facilities for changed and heavier particles are currently in operation worldwide, and most of them are represented by PT centers. Moreover, the numbers of PT facilities go to extend within the next five years, because some are under construction and in the planning stage. In conclusion, our results demonstrate the efficacy of PBCT in an in vivo experimental model of GBM and we highlight potentially exploitable marker to target in combination with PBCT to further increase its efficacy. We also featured the effects of sub-therapeutic radiation regimens using protons in combination with BPA in reducing tumor proliferation, highlighting the complex molecular network of interactions that PBCT induces in tumor cells, thus increasing the chances to overcome tumor resistance and recurrencies of such a deadly neoplasm.

## Methods

### Animal model

Experiments were performed on 8 weeks-old BALB/c Nude female mice (Charles River Laboratory), weighing 20 ± 3 g. Animals were housed in individual IVC cages with 3 subjects per cage, at constant temperature (23–25 °C) under a 12/12 h light/dark cycle with *ad libitum* access to food and water. All procedures were performed in accordance with the European Communities Council directive and Italian regulations (EEC Council 2010/63/EU and Italian D.Lgs. 26/2014). The project was approved by the Italian Ministry of Health (authorization number n.89/2020-PR). Efforts were made to replace, to reduce and to refine the use of laboratory animals. To avoid irrelevant suffering to treated mice, euthanasia was performed as soon as the final score was reached. The endpoint used to determine if animals should undergo euthanasia was reached when tumor lesions showed a dimension higher than 12 mm and/or weight loss more than 20%. To minimize suffering and mice distress, standard environmental enrichment of two nestles, a cardboard fun tunnel and one wooden chew block were provided.

To induce the heterotopic tumor, a total of 4 × 10^6^ U-87 MG cells (European Collection of Authenticated Cell Cultures, ECACC, Public Health England, UK) were injected into the subcutaneous flank of a total number of 37 mice. Animal health, body weight and specific clinical signs were monitored twice a week. At 20 days post graft (dpg) animals were randomly assigned to proton irradiated group, proton boron treated group or mock proton group, used as control group. Mice assigned to mock proton group followed superimposable protocol of irradiation with a dose of 0 Gy. At 30dpg mice were sacrificed and tumors were dissected out, stored at −80 °C for mRNA extraction or post-fixed, embedded in paraffin and sectioned using a microtome for immunohistochemistry analysis^52^.

### Live ultrasound and photoacoustic imaging of heterotopic GBM

Ultrasound and photoacoustic imaging (US-PAI) procedures were performed by using the Vevo 3100 LAZR-X Imaging Station (VisualSonics, Inc., Toronto, Canada) equipped with MX550D transducer operating in a range of 25–55 MHz frequency for high-resolution ultrasound imaging and a high-efficiency Vevo optical fiber with the necessary amplitude, depth, and sensitivity to achieve photoacoustic imaging co-registered with basic B-mode imaging. The main parameters of the Vevo LAZR laser system were as follow: peak energies of 45 mJ ± 5 mJ, with a pulse duration of 4–6 ns at adjustable wavelengths between 680 and 970 nm with 2 nm as step size. In order to perform all the procedures, mice were placed in an appropriate 2 L gas chamber filled with mixed O_2_ and isoflurane gas for veterinary use at a concentration of 4% induction and 2.5% maintenance. During anesthesia, a layer of water-soluble ophthalmic gel was put on mice’s eyes to protect and maintain the physiological ocular welfare. Mice were positioned in an appropriate heated platform, where 4 pad-mounted electrodes for ECG, temperature, and respiration data monitoring during the acquisition are embedded. Before each US-PAI acquisition, about 10 mm thick of hypoallergenic water-soluble US transmission gel was located on the tumor mass and on the transducer head, ensuring to eliminate air bubbles at any level into the gel layer.

For morphological and anatomical US imaging, *B-Mode* modality was used. The transmit center frequency was set at 40 MHz, and the acquisition was performed by adjusting the transducer positioning in real time, by identifying and centering the region of interest. In order to obtain an optimal imaging acquisition, the imaging focus depth was set at around 10 mm depth from the US/laser source.

To assess the amount of oxygenation and deoxygenation of the blood, an acquisition called “spectrum acquisition” was first performed using the full spectrum of wavelengths from 680 nm to 970 nm as a laser excitation that returns the spectral signal pattern of arterial and venous blood. Next, the Oxy – Hemo-mode was used, where the output wavelength of the PA laser during the acquisition was 750 nm and 850 nm, to isolate the distinct contributions of the signal due to the absorption of deoxygenated and oxygenated hemoglobin signals, respectively, obtaining a high-resolution parametric map of the oxygen saturation. Post-processing of the acquisitions is performed with Vevo Lab software (Fujifilm, VisualSonics, version 5.5.1), which allows reconstructing and analyzing the 3D volume of the area under investigation of which, thanks to the use of a motor with high-precision handling, axial and sequential 2D sections have acquired The analyses provided by VevoLAb which, involves the use of segmentation algorithms, allowed us to obtain the HemoMeaZure and OxyZated Tool volumetric measurements for the calculation and quantification of hemoglobin content and oxygen saturation and PAI intensity value. Regions of interest were plotted at the edges of the tumor, and this segmentation was repeated on each acquired frame, and 3D region measurements for both modalities were performed using specific measurement tools. The multi-Slice acquisition method and 3D post-processing reconstruction of the tumor mass allowed us to obtain transverse, sagittal, and coronal representations and to delineate and extrapolate the pathological mass from the context of the surrounding tissues.

### Proton irradiation and boron treatment

At 20dpg, mice were randomly assigned to mock proton, proton and proton boron groups. Mice treated with boron received a single intra-peritoneal injection of 500 mg/Kg of BPA in water. BPA was solubilized with a 10% molar excess of fructose in water at pH 9.5 and pH was then adjusted to 7.4 with HCl^28^. Mice treated with combined treatment have received boron 6 h before the proton treatment.

Proton irradiation was performed using a single sub-therapeutic dose of 10 Gy at 20dpg. The dose delivered to the biological sample was measured using transmission ionization chambers calibrated with a Markys-type ionization chamber placed at the isocenter. The dose rate was 15 Gy/min and the error on the released dose was found to be less than 3%^53,54^. The sub-therapeutic dose, in a single shot, was chosen to avoid side effects on skin and to obtain a visible treatment response on [^18^F] Fluorodeoxyglucose ([^18^F]-FDG) PET images and to perform the PET scans on all groups at the same time. Increasing the dose could have reduced different uptake between proton and proton boron groups. Reducing the dose could have reduced uptake different between mock proton and proton group^55^. Proton irradiation was performed at the Center for Adrotherapy Advanced Nuclear Applications (CATANA) proton therapy facility in National Institute for Nuclear Physics, Laboratori Nazionali del Sud (INFN-LNS) of Catania (Italy) using a passive proton beam line. In CATANA experimental hall, a fixed horizontal beam line was installed, and clinical proton beams can be delivered with a maximum energy of 62 MeV. A beam shaping system was used to obtain a uniform dose distribution at the isocenter. When proton beam reaches the experimental hall, it goes out in air and flies for three meters before hitting the target. Along its path, the beam is intercepted by various elements in order to obtain a flat transversal dose distribution at the isocenter. A dedicated animal holder system was used to ensure a precise and reproducible positioning. The lateral beam inhomogeneity was less than 2% at irradiation isocenter and, before irradiation, a check of beam flatness was performed using a motorized silicon detector^54^. A poly(methyl methacrylate) (PMMA) modulator wheel was used to create a SOBP featured by a plateau of 10.82 mm and a practical range of 11.68 mm, in water. Moreover, a range shifter was used to obtain a uniform dose distribution from entrance because the animal tumors were under the skin. The uncertainty in the determination of the absorbed dose during the beam calibration process was evaluated to be less than 3%. The field size was shaped using a 6.3 mm thick brass in-house-made collimator with a disc opening of 1 cm in diameter. Mice were sacrificed at 30dpg and isolated tumors were collected for subsequent analysis.

### Micro-positron emission tomography/computed tomography imaging

μPET-CT imaging with clinical grade [^18^F]-FDG, prepared accordingly to European Pharmacopeia VIII Edition with a radiochemical purity >99%, was used to assess hypermetabolic tumors in mice.

[^18^F]-FDG at 4 MBq was intravenously injected in a total volume of 100 μL per 20 g of body weight normalized over the body mass index. All μPET-CT scanning were performed on the whole mouse body, a CT scan (45 kVp, 400 uA), and a 3D whole-body PET scan (5 min/bed position), using the Albira Si Bruker at the Center for Advanced in vivo Preclinical Research (CAPiR) facility - University of Catania. PET images were reconstructed using the maximum-likelihood expectation maximization (MLEM) GPU algorithm with corrections for random, scatter and attenuation. μPET-CT image volume consisted of 160 × 160 voxels of 0.5 × 0.5 × 0.5 mm^3^ size.

For quantification of hypermetabolic μPET tumors, an innovative and operator-independent segmentation method was used for the identification of the metabolic tumor volume (MTV)^56^. This algorithm was adapted to preclinical images to reconstruct the 3D shape of the tumor. Briefly, the algorithm converts Digital Imaging and Communications in Medicine (DICOM) images into Standardized Uptake Value (SUV) images^57^. The SUV is the most commonly used semi-quantitative parameter in nuclear medicine, and calculated using Eq. 1.

1 $${SUV}=\frac{{radioactivity}\,{of}\,{the}\,{tumor}}{{injected}\,{radioactivity}}\times {body}\,{weight}$$

After converting the PET image to SUV images, the user identified the tumor-containing region on a single PET slice. Subsequently, the algorithm automatically identified the slice enclosing the maximum SUV within the tumor volume and generates, through the region growing method, a rough and user-independent contour surrounding the tumor, as previously described^58^.

This contour was then used to initialize an active surface that evolved directly in the 3D space, leveraging cross-slice information that could not be exploited in 2D implementations. Without any specific stopping condition, the active surface naturally reached a stable topology once convergence was achieved. By construction, the MTV was independent from the operator that initially identified the tumor-containing region, making the result robust and repeatable^56^. At this stage, SUVs were extracted from MTVs and were corrected to overcome the partial volume effect (PVE). Given that PVE significantly affects the estimation of semi-quantitative parameters, a PVE correction method based on Recovery Coefficients (RC) as function of measured lesion-to-background ratio and measured lesion volume was adapted to preclinical images. SUVs were corrected by dividing them by the corresponding RC to the MTV sphere-equivalent diameter (d) ranging from 1 mm to 5 mm (Table 1). For sphere-equivalent diameters greater than 5 mm, no corrections were adopted as the PVE was considered irrelevant.

**Table 1:** Recovery coefficients per MTV sphere-equivalent diameter.

Diameter	1 mm	2 mm	3 mm	4 mm	5 mm
Recovery Coefficient	0.04	0.19	0.41	0.54	0.70

Finally, the total lesion glycolysis (TLG) was calculated as the product of the SUV_mean_ (i.e. the mean SUV within MTV) and the MTV to provide estimation of both volumetric and metabolic information. TLG is used as index for lesion malignancy differentiation and monitoring of disease and response to treatment.

SUV variations in sequential PET scans was normalized to baseline and expressed as percentage of variation (Δ) according to the equation defined by the European Organization for Research and Treatment of Cancer (EORTC, Eq. 2)^59^.

2 $$\Delta {SUV}=\frac{{SUV}{post}-{SUV}{pre}}{{SUV}{pre}}\times 100$$

Based on this value, it is possible to distinguish between progressive disease if $\Delta {SUV}$>25%, stable disease if −15%<ΔSUV < 25%, and partial response if ΔSUV < −15%, as previously reported^60^. In the same way, ΔTLG and ΔMTV were calculated.

### Immunohistochemistry and immunofluorescence

Sample slides were dewaxed in xylene, hydrated using decreasing concentration of ethanol in water (100%, 75%, 50%, 25%) and then water for 5 min at room temperature.

For immunohistochemical analysis, rehydrated samples were then incubated for 30 min in 0.3% H_2_O_2_ to quench endogenous peroxidase activity, then rinsed for 20 min with PBS. Sections were subjected to a protocol of antigen retrieval heating (5 min x 3) in capped polypropylene slide-holders with citrate buffer (10 mM citric acid, 0.05% Tween 20, pH 6.0), using a microwave (750 W). To reduce non-specific immunoreactivity due to endogenous biotin, sections were pretreated with 10 mg/mL of ovalbumin in PBS followed by 0.2% biotin in PBS, each for 15 min at room temperature. Sections were then incubated with mouse monoclonal anti-KI67 antibody (Agilent Cat# GA626, RRID:AB_2687921), rabbit polyclonal anti-cleaved caspase-3 antibody (Abcam Cat# ab2302, RRID:AB_302962). Then, samples were washed in 0.3% Triton in PBS three times for 5 min and samples were incubated with appropriate secondary biotinylated antibody for 30 min at room temperature, followed by the avidin–biotin–peroxidase complex (Vector Laboratories) for 30 min at room temperature. The immunoreaction was visualized by incubating the sections for 4 min in a 0.1% 3,30-diaminobenzidine (DAB) and 0.02% hydrogen peroxide solution (DAB substrate kit, Vector Laboratories). Nuclei were counterstained with Mayer’s hematoxylin (Histolab Products AB) mounted in GVA mounting medium (Zymed Laboratories). Pictures were acquired using a Zeiss Axioplan light microscope. Hematoxylin and eosin-stained sections were used for apoptotic cells and mitotic index evaluation. The total number of apoptotic cells, mitotic cells and Ki67 positive nuclei were assessed per 10 high power fields (HPFs) at 40X magnification using a Zeiss Axioplan light microscope. For caspase quantification, staining intensity score (IS) was graded on a 0–3 scale, according to the following assessments: no detectable staining=0, weak staining = 1, moderate staining = 2, strong staining = 3. Moreover, the percentage of immunopositivity cells (Extent Score, ES) was scored in five categories: <5% (0); 5–30% (1); 31–50% (2); 51–75% (3), and >75% (4). Counting was performed at 200X magnification. In addition, staining intensity was multiplied by the percentage of positive cells to obtain the intensity reactivity score (IRS).

For immunofluorescence, GBM sections were deparaffinized and rehydrated as described above. To investigate autophagy and mitophagy, slides were washed in 0.3% Triton X-100 in PBS, two times for 5 min at room temperature. Then sections were blocked with blocking solution (10% normal goat serum (NGS)) for 1 h at room temperature in a humidity chamber. Sections were labeled with phospho-ATM (Ser1981) mouse monoclonal antibody (1:200, Invitrogen Cat# MA1-2020, RRID:AB_1086244) or with 10 µM MitoTracker Red CMXRos probe (M7512, Thermo Fisher Scientific, Rodano, Milan, Italy) and LC3-II rabbit polyclonal antibody (1:100, Sigma-Aldrich Cat# L7543, RRID:AB_796155) with the own appropriate dilution in incubating solution (1% NGS in 0.3% Triton X-100 in PBS). On the following day, after three washes with 0.3% Triton X-100 in PBS, slides were incubated for 1 h at room temperature with goat polyclonal anti-rabbit (Alexa Fluor 488, Molecular Probes, Cat#A-11008, RRID: AB_143165). Nuclei were counterstained with 4,6-diamidino-2-phenylindole (Dapi, Invitrogen, Cat. No. D1306, 1:1000) for 3 min at room temperature and then mounted with BrightMount (Abcam, Cat#ab103746). Digital images were acquired and quantified using a Leica TCS SP8 confocal microscope. For quantification of LC3B mean fluorescence intensity (MFI), and mitochondrial signal colocalization *n* ≥ 5 regions of interest per *n* ≥ 3 sections per animal were analyzed and quantified by operators blinded to the treatment using ImageJ v. 2.1.0/1.53c (Fiji) software.

### RNA sequencing

Total RNA was extracted from tissue samples using Trizol and the RNeasy mini kit (Invitrogen) and RNA concentration and purity were determined using a Nanodrop ND-1000 (Thermo Scientific Open Biosystems, Lafayette, CO, USA). Samples with an RNA integrity number (RIN) value of 10, assessed by using a Bioanalyzer 2100 (Agilent Technologies, Santa Clara, CA, USA), were used for further analyses. Thus, RNA samples were analyzed according to the RNA sequencing Gene Expression Analysis protocol (BMR genomics, Padova, Italy). Enrichment of mRNA from total RNA was conducted using poly-T oligo-attached magnetic beads. The mRNA fragments were then randomly broken into short fragments that were used as a template to synthesize cDNA for the construction of libraries for sequencing. Transcripts quantification and sequencing, as well as quality control of libraries, were performed using Illumina NovaSeq 6000 platform (Illumina Netherlands, The Netherlands). Finally, we studied biological pathways regulated by the genes belonging to the differentially expressed gene lists obtained by RNA-seq analyses, firstly using the Database for Annotation, Visualization and Integrated Discovery (DAVID) network building tool (https://david.ncifcrf.gov/tools.jsp) which provides a comprehensive set of functional annotations for investigators to study the biological content captured by high-throughput technologies and secondly by using the QIAGEN Ingenuity Pathway Analysis (IPA) (QIAGEN Aarhus Prismet, Aarhus C, Denmark) to confirm our assumptions. The use of different pathway analysis tools, formed by multiple experimental and bibliographic datasets, could enrich data because analyzed using different reading ways. These enrichment approaches are often chosen to interpret a great amount of data such as those provided by Next Generation Sequencing experiments.

### Statistics and reproducibility

All tests were performed in GraphPad Prism (version 5.00 for Mac, GraphPad Software) or Rstudio (version 1.0.153, Rstudio Inc., Boston, USA). Data were tested for normality using a Shapiro–Wilk normality test and subsequently assessed for homogeneity of variance. Data that passed both tests were further analyzed by two-tailed unpaired Student’s *t* test for comparison of *n* = 2 groups. Comparisons of *n* > 2 groups were performed using a one-way ANOVA and Holm–Sidak’s multiple comparisons test. For data that did not pass normality test, Kruskal–Wallis test was used for comparisons between groups. The sample size for each experiment is reported in Supplementary data 2. For all statistical tests, *p* values < 0.05 were considered statistically significant.

### Reporting summary

Further information on research design is available in the Nature Portfolio Reporting Summary linked to this article.

## Supplementary information

Supplementary Information

Description of Additional Supplementary Files

Supplementary Data 1

Supplementary Data 2

Reporting Summary

## Data Availability

Data supporting the findings of this study are available within the article and source data are provided in supplementary information files (Supplementary Data 2). The Bioproject (PRJNA943942), Sequence Read Archive (SRA submission: SUB12943079), and BioSamples accessions data, were deposited at National Center for Biotechnology Information. Any remaining information can be obtained from the corresponding author upon reasonable request.
