# Supplementary material for: Proton boron capture therapy (PBCT) induces cell death and mitophagy in a heterotopic glioblastoma model
Source: Commun Biol. 2023 Apr 8;6:388. doi: 10.1038/s42003-023-04770-w (PMC10082834; doi:10.1038/s42003-023-04770-w)
Supplement: Supplementary file 1 — Supplementary Information [file 42003_2023_4770_MOESM1_ESM.pdf]

Supplementary material

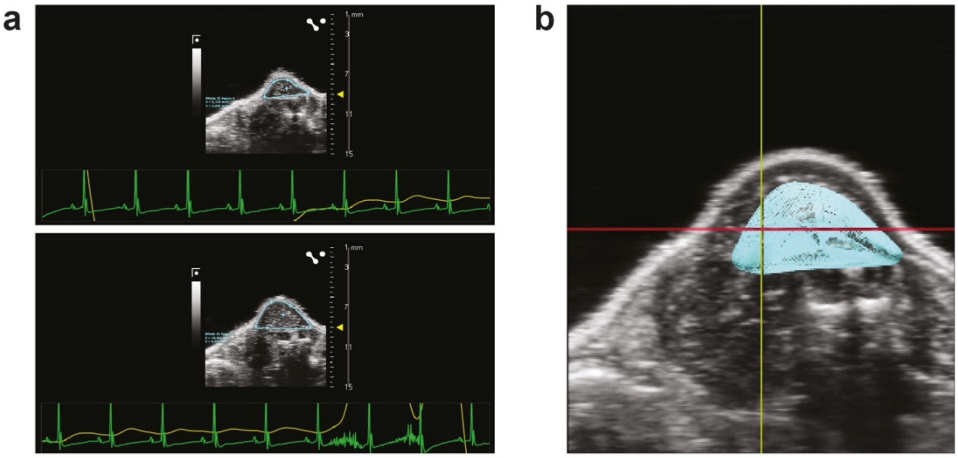

Supplementary Figure 1. Segmentation of tumor mass for volume measurement and 3D image reconstruction. a) To create a 3D region, the first 2D frame was selected as the starting point and a second region was segmented in a next frame. b) Vevo LAB Software automatically draws contours on every slice between the two regions using specific algorithm. After the selection of each frame, the volume is displayed in 3D and measures are reported in mm^3.

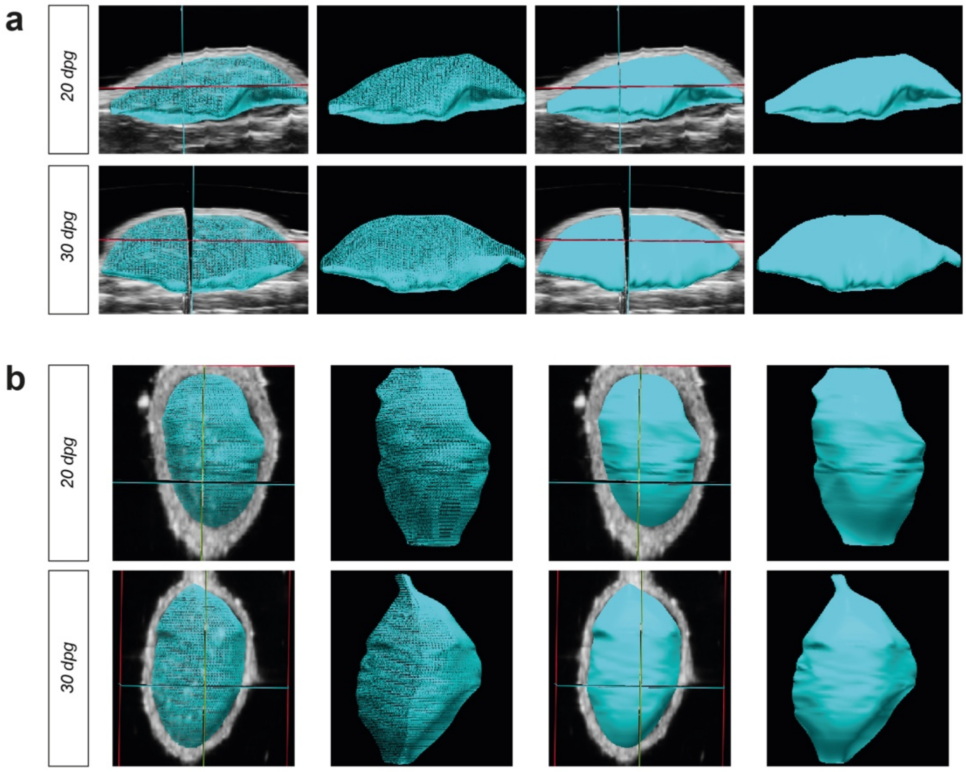

Supplementary Figure 2. Representative images for tumor mass 3D reconstruction 20 and 30 d.p.g. a-b) Each series of sagittal (a) and coronal (b) views are represented in both meshed and overlayed surface modality with and without the x, y, and z axe**.**

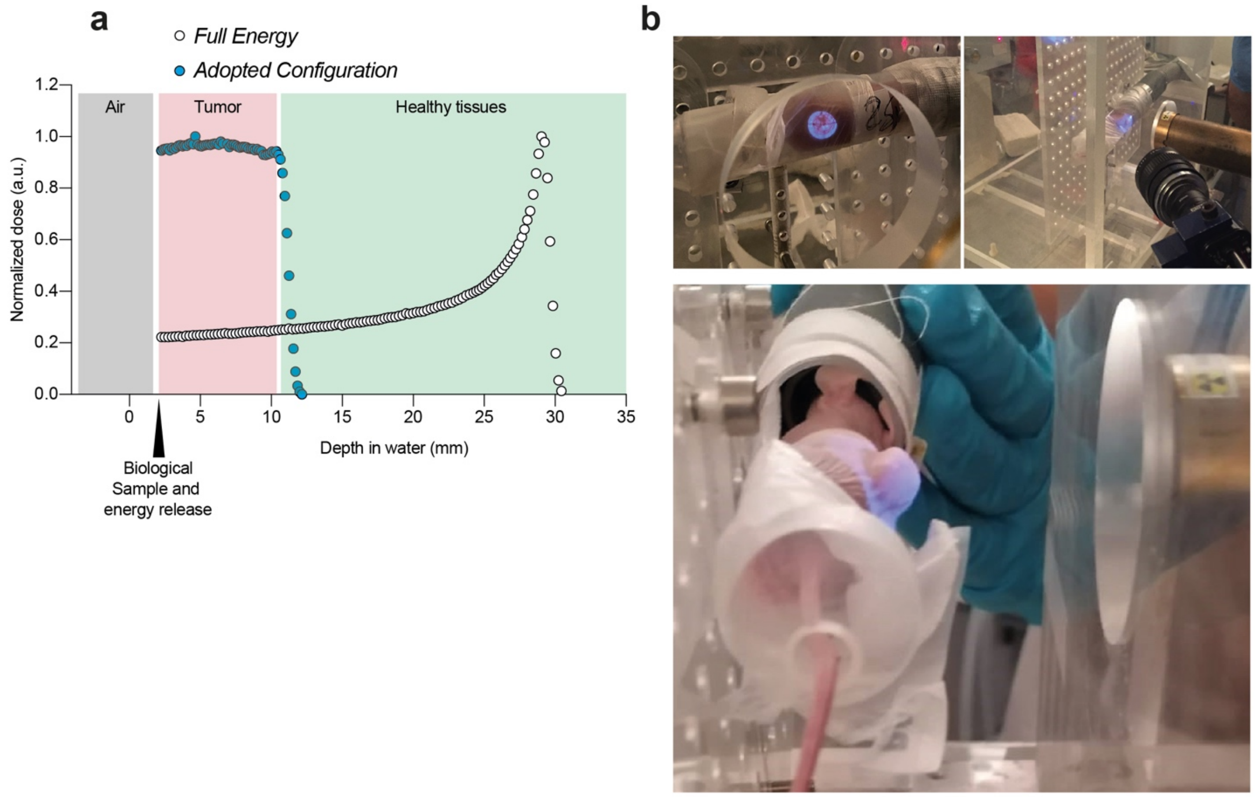

Supplementary Figure 3. Dosimetric proton profile and proton beam irradiation on heterotopic GBM mouse model. a) Experimental depth dose distribution of protons is represented for the SOBP creation (blue dots, adopted configuration) versus typical Bragg peak (white dots, full energy). b) Light field allows the correct position of collimator at the specific site to be irradiated; a video camera allows the remote viewing to ensure that no movement occurs during irradiation.

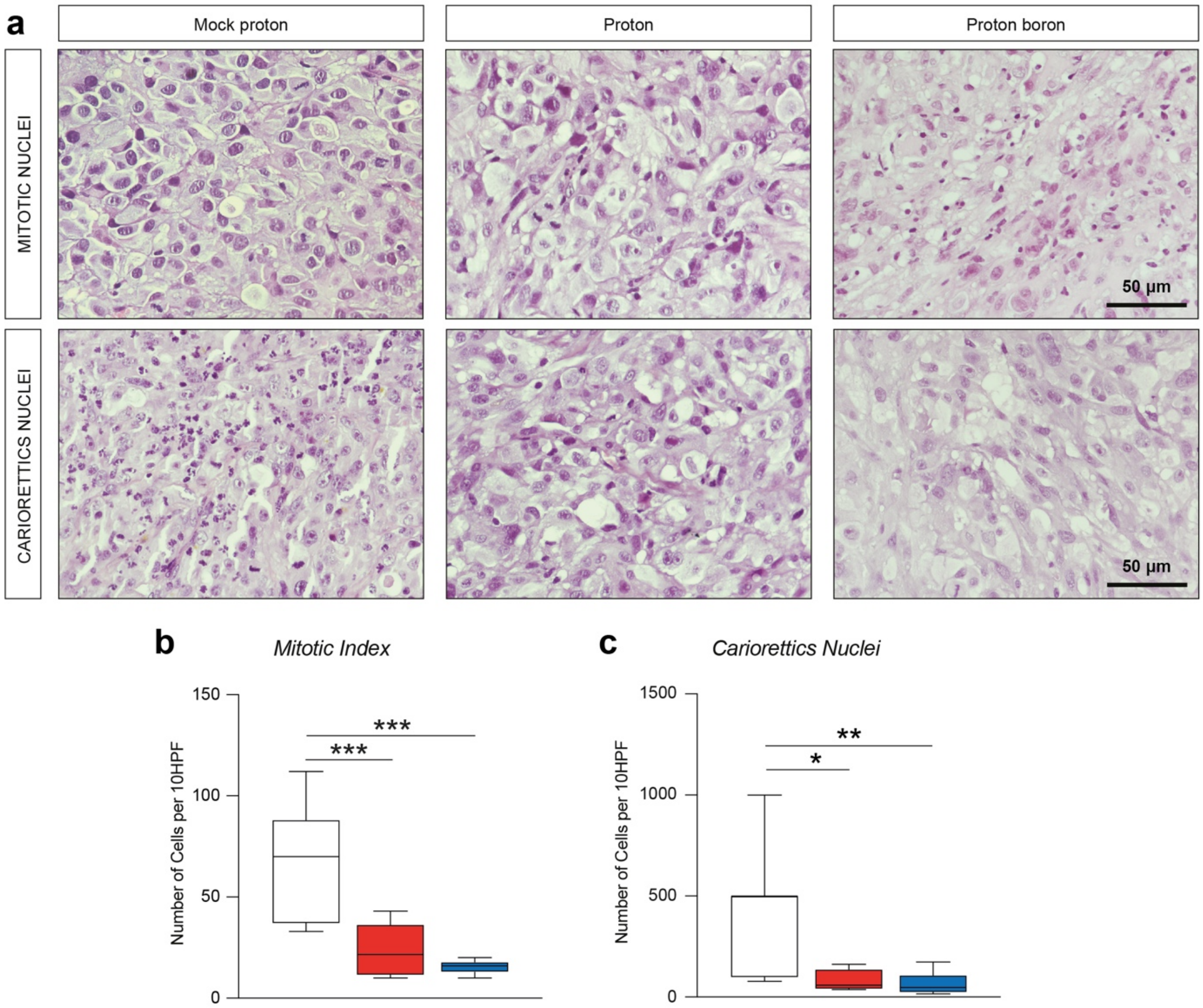

Supplementary Figure 4. PBCT and proton irradiation induces comparable effects for mitosis and nuclear integrity. a) Representative images of mitotic and cariorettics nuclei in GBM tumor sections derived from mock proton, proton-treated and proton boron-treated mice. b-c) Quantification of mitotic index (b) and cariorettics nuclei (c) in GBM tumor sections derived from mock proton (white boxes), proton-treated (red boxes) and proton boron-treated (blue boxes) mice. *p-value < 0.05, **p-value < 0.01 and ***p-value < 0.001.
