# Supplementary material for: Proton boron capture therapy (PBCT) induces cell death and mitophagy in a heterotopic glioblastoma model
Source: Commun Biol. 2023 Apr 8;6:388. doi: 10.1038/s42003-023-04770-w (PMC10082834; doi:10.1038/s42003-023-04770-w)
Supplement: Supplementary file 2 — Description of Additional Supplementary Files [file 42003_2023_4770_MOESM2_ESM.pdf]

# Description of Additional Supplementary Files

**File name:** Supplementary Data 1

**Description:** Comparative differential gene expression analysis of the proton boron treated group versus proton and DAVID pathways analysis of the 138 differentially expressed genes of the proton boron treated group versus proton
