# Supplementary material for: Proton boron capture therapy (PBCT) induces cell death and mitophagy in a heterotopic glioblastoma model
Source: Commun Biol. 2023 Apr 8;6:388. doi: 10.1038/s42003-023-04770-w (PMC10082834; doi:10.1038/s42003-023-04770-w)
Supplement: Supplementary file 5 — Reporting Summary [file 42003_2023_4770_MOESM5_ESM.pdf]

nature portfolio

Corresponding author(s): Giorgio Russo and Rosalba Parenti

Last updated by author(s): Mar 15, 2023

Reporting Summary

Nature Portfolio wishes to improve the reproducibility of the work that we publish. This form provides structure for consistency and transparency in reporting. For further information on Nature Portfolio policies, see our Editorial Policies and the Editorial Policy Checklist.

Statistics

For all statistical analyses, confirm that the following items are present in the figure legend, table legend, main text, or Methods section.

n/a	Confirmed
☐	☒ The exact sample size (*n*) for each experimental group/condition, given as a discrete number and unit of measurement
☐	☒ A statement on whether measurements were taken from distinct samples or whether the same sample was measured repeatedly
☐	☒ The statistical test(s) used AND whether they are one- or two-sided<br>*Only common tests should be described solely by name; describe more complex techniques in the Methods section.*
☐	☒ A description of all covariates tested
☐	☒ A description of any assumptions or corrections, such as tests of normality and adjustment for multiple comparisons
☐	☒ A full description of the statistical parameters including central tendency (e.g. means) or other basic estimates (e.g. regression coefficient) AND variation (e.g. standard deviation) or associated estimates of uncertainty (e.g. confidence intervals)
☐	☒ For null hypothesis testing, the test statistic (e.g. *F*, *t*, *r*) with confidence intervals, effect sizes, degrees of freedom and *P* value noted<br>*Give P values as exact values whenever suitable.*
☐	☒ For Bayesian analysis, information on the choice of priors and Markov chain Monte Carlo settings
☐	☒ For hierarchical and complex designs, identification of the appropriate level for tests and full reporting of outcomes
☐	☒ Estimates of effect sizes (e.g. Cohen's *d*, Pearson's *r*), indicating how they were calculated

Our web collection on statistics for biologists contains articles on many of the points above.

Software and code

Policy information about availability of computer code

Data collection	Vevo 3100 LAZR-X Imaging Station and VevoLAb (VisualSonics, Inc., Toronto, Canada);
Data analysis	Vevo 3100 LAZR-X Imaging Station and VevoLAb (VisualSonics, Inc., Toronto, Canada); GraphPad Prism (version 5.00 for Mac, GraphPad Software); ImageJ v. 2.1.0/1.53c (Fiji).

For manuscripts utilizing custom algorithms or software that are central to the research but not yet described in published literature, software must be made available to editors and reviewers. We strongly encourage code deposition in a community repository (e.g. GitHub). See the Nature Portfolio guidelines for submitting code & software for further information.

Data

Policy information about availability of data

All manuscripts must include a data availability statement. This statement should provide the following information, where applicable:

- Accession codes, unique identifiers, or web links for publicly available datasets
- A description of any restrictions on data availability
- For clinical datasets or third party data, please ensure that the statement adheres to our policy

Data supporting the findings of this study are available within the article, its supplementary information files, and from the corresponding authors upon reasonable request. The Bioproject (PRJNA943942), Sequence Read Archive (SRA submission: SUB12943079), and BioSamples accessions data, were deposited at National Center for Biotechnology Information. Source data are provided with this paper.

Human research participants

Policy information about studies involving human research participants and Sex and Gender in Research.

Reporting on sex and gender	NA
Population characteristics	NA
Recruitment	NA
Ethics oversight	NA

Note that full information on the approval of the study protocol must also be provided in the manuscript.

Field-specific reporting

Please select the one below that is the best fit for your research. If you are not sure, read the appropriate sections before making your selection.

☒ Life sciences ☐ Behavioural & social sciences ☐ Ecological, evolutionary & environmental sciences

For a reference copy of the document with all sections, see nature.com/documents/nr-reporting-summary-flat.pdf

Life sciences study design

All studies must disclose on these points even when the disclosure is negative.

Sample size	Our sample sizes were similar to those reported in previous publications. Page #4-11 of the manuscript.
Data exclusions	Mice were randomly assigned to experimental groups. No data points or animals were excluded from the analysis.
Replication	Page #4-11 of the manuscript.
Randomization	Page #4-11 of the manuscript.
Blinding	Samples analysis were analyzed and quantified by operators blinded to the treatment.

Reporting for specific materials, systems and methods

We require information from authors about some types of materials, experimental systems and methods used in many studies. Here, indicate whether each material, system or method listed is relevant to your study. If you are not sure if a list item applies to your research, read the appropriate section before selecting a response.

Materials & experimental systems

n/a	Involved in the study	
☐	☒	Antibodies
☐	☒	Eukaryotic cell lines
☒	☐	Palaeontology and archaeology
☐	☒	Animals and other organisms
☒	☐	Clinical data
☒	☐	Dual use research of concern

Methods

n/a	Involved in the study	
☒	☐	ChIP-seq
☒	☐	Flow cytometry
☒	☐	MRI-based neuroimaging

Antibodies

Antibodies used	Page #4-11 of the manuscript.
Validation	Page #4-11 of the manuscript.

Eukaryotic cell lines

Policy information about cell lines and Sex and Gender in Research

Cell line source(s)	ECACC
Authentication	Cell line used was not authenticated.
Mycoplasma contamination	Cell line used was not tested for mycoplasma contamination.
Commonly misidentified lines (See ICLAC register)	None.

Animals and other research organisms

Policy information about studies involving animals; ARRIVE guidelines recommended for reporting animal research, and Sex and Gender in Research

Laboratory animals	8 weeks-old BALB/c Nude mice.
Wild animals	The study did not involve wild animals.
Reporting on sex	Female animals were used in this study. No sex-related issue and findings are applicable to both sex.
Field-collected samples	The study did not involve samples collected from the field.
Ethics oversight	All experiments were performed in accordance with the principle of the Basel Declaration as well as to the European Union Council directive and Italian regulations (2010/63/EU and Italian D. Lgs. no. 26/2014). All efforts were made to replace, reduce and refine the use of laboratory animals. Moreover, the study was conducted in accordance with the recommendations of the local committee for animal welfare (OPBA, University of Catania, Via Santa Sofia 97, Catania, Italy); the protocol was approved by OPBA and by the Italian Ministry of Health.

Note that full information on the approval of the study protocol must also be provided in the manuscript.
